# Supplementary figures and images for: A Comprehensive Characterization of Small RNA Profiles by Massively Parallel Sequencing in Six Forensic Body Fluids/Tissue
Source: Genes (Basel). 2022 Aug 25;13(9):1530. doi: 10.3390/genes13091530 (PMC9498867; doi:10.3390/genes13091530)

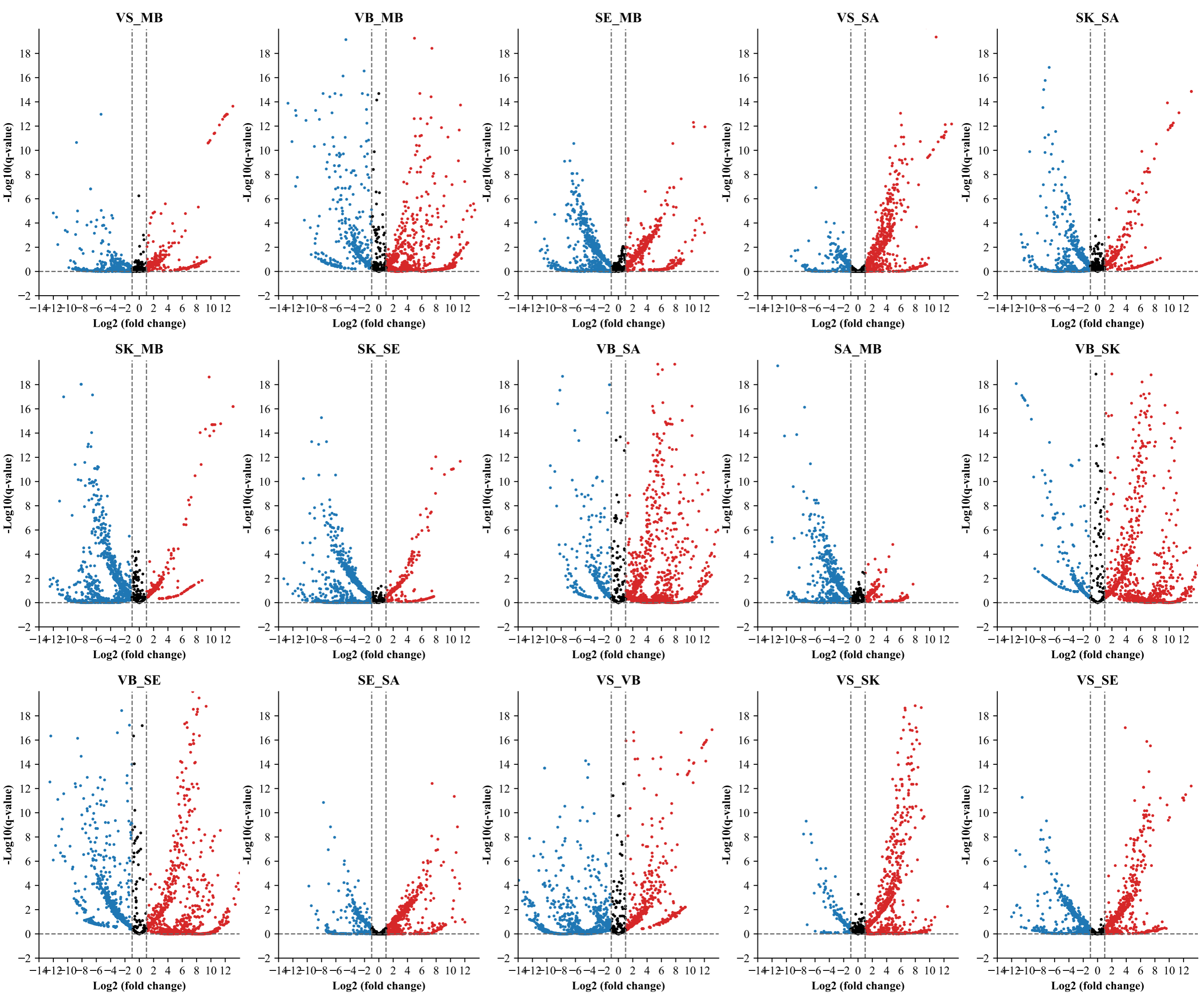

Supplement: Supplementary file 1 [file genes-13-01530-s001.zip › supplementary_updated/Figure_S2.pdf]

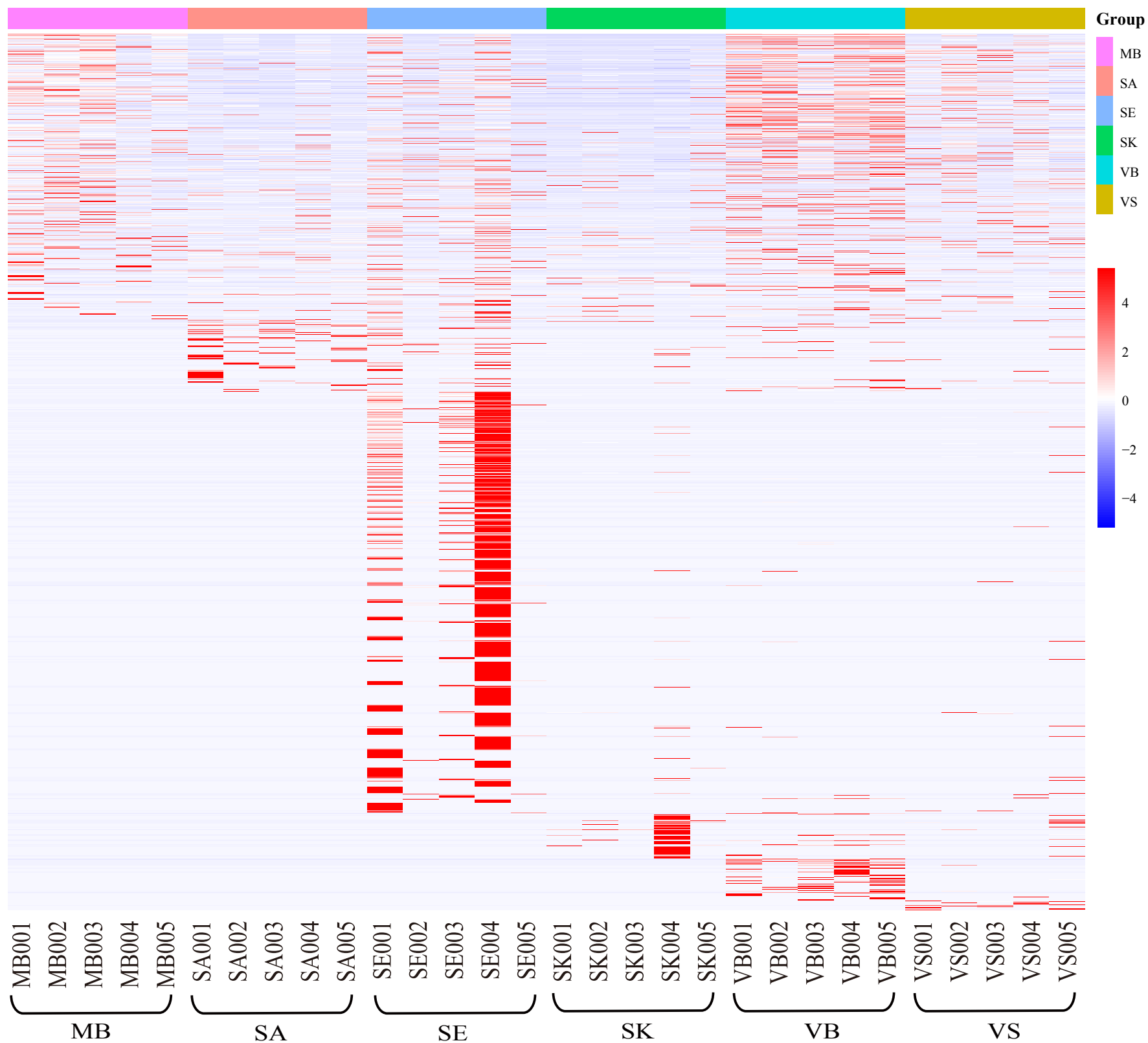

Supplement: Supplementary file 1 [file genes-13-01530-s001.zip › supplementary_updated/Figure_S1.pdf]
